# Glioma-related epilepsy following low-grade glioma surgery

**DOI:** 10.1093/noajnl/vdae127

**Published:** 2024-07-20

**Authors:** Amir Saam Youshani, Calvin Heal, Jing X Lee, Michael Younis, Rajiv Mohanraj, Helen Maye, Matthew Bailey, David Coope, Pietro I D’Urso, Konstantina Karabatsou

**Affiliations:** Department of Neurosurgery, Salford Royal NHS Foundation Trust, Greater Manchester Neurosciences Centre, Manchester, UK; Division of Neurosciences, School of Biological Sciences, Faculty of Biology, Medicine and Health, University of Manchester, Manchester Academic Health Sciences Centre, Manchester, UK; Division of Population Health, Health Services and Primary Care, School of Health Sciences, Faculty of Biology, Medicine and Health, Manchester Academic Health Sciences Centre, Manchester, UK; Department of Neurosurgery, Salford Royal NHS Foundation Trust, Greater Manchester Neurosciences Centre, Manchester, UK; Manchester Medical School, School of Medical Sciences, University of Manchester, Manchester, UK; Department of Neurology, Salford Royal NHS Foundation Trust, Greater Manchester Neurosciences Centre, Manchester, UK; Department of Neurosurgery, Salford Royal NHS Foundation Trust, Greater Manchester Neurosciences Centre, Manchester, UK; Department of Neurosurgery, Salford Royal NHS Foundation Trust, Greater Manchester Neurosciences Centre, Manchester, UK; Department of Neurosurgery, Salford Royal NHS Foundation Trust, Greater Manchester Neurosciences Centre, Manchester, UK; Division of Neurosciences, School of Biological Sciences, Faculty of Biology, Medicine and Health, University of Manchester, Manchester Academic Health Sciences Centre, Manchester, UK; Department of Neurosurgery, Salford Royal NHS Foundation Trust, Greater Manchester Neurosciences Centre, Manchester, UK; Department of Neurosurgery, Salford Royal NHS Foundation Trust, Greater Manchester Neurosciences Centre, Manchester, UK

**Keywords:** anti-seizure medication, epilepsy, glioma, low-grade glioma, seizure

## Abstract

**Background:**

Epileptic seizures commonly burden low-grade glioma (LGG) patients and negatively impact quality of life, neurocognition, and general patient health. Anti-seizure medications (ASMs) are used to manage seizures but can result in undesired side effects. Our aim was to report our experience in epilepsy in one of the largest case series of LGG patients (reclassified in accordance with the WHO 2021 classification). Furthermore, we evaluate our postoperative seizure frequency difference between LGG patients who use preoperative ASMs and ones with no ASMs.

**Methods:**

Data were retrospectively collected from Salford Royal Hospital electronic records and Neuro-Oncology database from 2006 to 2022. Descriptive statistics were performed for demographic analysis, while multivariable analysis was used to determine postoperative seizure-free outcomes.

**Results:**

In total, 257 operations were performed on 206 patients. Postoperatively, 114 patients suffered from seizures, and approximately 45.2% of patients developed seizures at 3–12 months postsurgery, with the odds higher in patients on preoperative ASMs. There was no evidence to suggest a higher postoperative seizure rate in patients undergoing awake craniotomy versus general anesthetic. The extent of resection (EOR) was inversely related to seizure failure, with gross-total resection showing a statistically significant reduction in seizures in comparison to all other surgical resections.

**Conclusions:**

In our experience, there is no evidence to suggest a reduced postoperative seizure outcome when prescribing preoperative ASMs. EOR is an independent prognosticator for postoperative seizure failure with all other variables demonstrating nonsignificance. Overall, a larger study can investigate the role of ASMs in LGG in greater detail.

**Key Points**

- A large case series of WHO grade 2 low-grade glioma patients in accordance with WHO 2021 classification.
- Gross-total resection shows a significantly reduced postoperative seizure burden.
- Awake craniotomy does not significantly contribute to postoperative seizure frequency.

**Importance of the Study**

To date, studies have shown the postoperative seizure outcome using the WHO classification 2016. We have histologically reclassified all cases in accordance with the 2021 update. In one of the largest series of patients, we report our postoperative seizure outcomes. In addition, we evaluate a cohort of patients using anti-seizure medications (ASMs) versus those without to determine the prophylactic effect of preoperative ASMs. A multivariate analysis was performed for awake versus general anesthetic surgery, as well as reviewing the impact of extent of resection on postoperative seizures. Our results support the rationale of maximal surgical resection with an awake surgery when possible, but suggest a larger study with a randomized controlled trial to determine the rationale of preoperative ASM. The findings from this study will be applicable to neurosurgeons, neurologists, oncologists, and allied health professionals in the multidisciplinary team.

Epileptic seizures occur in 60%–85% of patients diagnosed with low-grade gliomas (LGGs).^1–3^ Seizure type and frequency vary in accordance with tumor characteristics. Typically, lobar location, cortical versus noncortical lesions, tumor type, and grade are all suggested risk factors to develop tumor-related seizures.^2^ Such epileptic instability can lead to refractory epilepsy in up to 15% of cases despite surgical tumor resection and oncological treatment.^4,5^

Both diffuse astrocytoma and oligodendroglioma are considered as epileptogenic intrinsic brain tumors. LGGs infiltrate the cortex as well as the subcortical white matter and concurrently dysregulate the excitatory–inhibitory balance in the peritumoral surrounding brain.^6^ Molecular pathways described to be involved with intrinsic epileptogenicity include: (RAS)/mitogen-activated protein kinase/extracellular signal-regulated kinase (MAPK/ERK) and phosphatidylinositol-3-kinase/protein kinase B/mammalian target of rapamycin (P13K/AKT/mTOR) pathways and more recently BRAFv600E mutation.^6,7^ Furthermore, isocitrate dehydrogenase (IDH) mutations convert alpha-ketoglutarate to D-2-hydroglutarate (D-2-HG), resulting in lowered seizure thresholds.^8^ The inherent heterogeneity of LGGs complicates symptomatic management of seizures further, with the overall pathogenic mechanisms poorly understood.^6^ The complexity at the cellular level translates to the difficulty encountered when managing seizures, which requires a combination of different anti-seizure medications (ASMs). However, medical management with ASMs is not without consequences and can result in adverse effects that impair quality of life. In addition, ASMs, especially those that induce hepatic microsomal enzymes, may directly impact oncological treatment by interacting with critical chemotherapeutic agents.^9^

No overall consensus exists regarding the use of ASMs and the type of drug to be used perioperatively. Historically, a double-blinded randomized controlled trial showed a significant decrease in early postsurgical seizures treated with phenytoin prophylaxis.^10^ However, a Cochrane review demonstrated no difference in postcraniotomy seizure incidence with or without anticonvulsants.^11^ The Society for Neuro-Oncology (SNO) and European Association of Neuro-Oncology (EANO) guidelines currently discourage the routine use of prophylactic ASMs in patients undergoing craniotomy for tumor resection if there are no previous seizure episodes.^12^ However, some surgeons prefer to load on prophylactic ASMs 24 hours presurgery, due to the risks of intraoperative seizures, which can reach up to 12.6% during awake craniotomies.^13^

In patients with glioma-related epilepsy (GRE), a recent systematic review showed that levetiracetam had the highest efficacy in producing seizure freedom at 6 months.^14^ The studies were however noted to be of low quality and likely did not include several other oral ASMs commonly used in clinical practice. A large-scale randomized control trial in the United Kingdom has shown that lamotrigine is superior to levetiracetam in focal epilepsies.^15^ However, the use and choice of agent continues to rely on surgeon’s/neurologist’s preference and experience. Importantly, a greater tumor resection is correlated with lower seizure frequency.^16,17^ Other prognosticators would prove beneficial to prevent seizure occurrence; however, extrapolating definitive conclusions from current studies is limited because of: heterogenous populations used within different studies, a changing World Health Organization (WHO) classification that has led to reclassifying LGGs and methodological differences resulting in further variability. In addition, the majority of studies only focus on early postoperative seizures and not the longer-term sequelae occurring during tumor evolution.

In this retrospective study, we report our center's experience in one of the largest case series of LGG patients and the postoperative GRE outcomes. In addition, we have reclassified all patient samples histology in accordance with the WHO 2021 classification and evaluated our findings. Thus far, very few studies have shown a large case series of patients with the updated WHO classification.^18^ Finally, our aim was to investigate the differences between 3 groups of patients: (i) patients taking ASMs for presurgery seizures, (ii) prophylactic ASMs used from the day of surgery, and (iii) no ASMs used before or after surgery. The objective was to determine the benefits of ASMs prior to surgery and the different surgical techniques used for seizure outcome at an early and later postoperative period.

## Methods

### Data Source and Collection

Data acquisition was approved by the Salford Royal NHS Foundation Trust Audit Department and the Neuro-Oncology Tumour Board.

We searched the Neuro-Oncology database for cases of IDH-mutant LGG from May 2006 to May 2022 and included: oligodendroglioma, diffuse astrocytoma, and oligoastrocytoma. All cases were rereviewed in accordance with the up-to-date histological WHO classification 2021, and only WHO grade 2 oligodendroglioma and astrocytoma pathologies were included in this study.^19^ Thus, cases previously diagnosed as oligoastrocytoma were reclassified into either grade 2 diffuse astrocytoma or oligodendroglioma. Cases that did not meet the diagnostic criteria of LGG were subsequently excluded from the study.

Inclusion criteria consisted of (i) patients over 18 years of age at diagnosis, (ii) histopathological diagnosis of WHO grade 2 gliomas, and (iii) a minimum of 12-month follow-up period.

Information collected from the Neuro-Oncology database consisted of age, gender, initial presenting symptom, presence of epileptic seizure at the time of diagnosis, type of ASM used (established for more than 2 weeks or prophylactic), side effects of ASMs, and surgical technique (awake versus general anesthesia procedure).

Imaging characteristics analyzed included tumor side, tumor main anatomical location, and extent of surgical resection based on preoperative and immediate postoperative MRI imaging on T2-weighted FLAIR sequences (biopsy versus partial resection versus near-total versus gross-total resection).

Surgical extent of resection (EOR) was scored in accordance with Karschnia et al.: gross-total resection (GTR), 100%; near-total resection (NTR), 90%–100%; subtotal resection (STR), <90%; and biopsy, <50%.^20^ EOR was calculated by the neuroradiologist and reviewed in the multidisciplinary team (MDT) outcome.

### Endpoints

We evaluated the prevalence of seizure occurrence at presentation, immediate postoperative period (<15 days from surgery), early postoperative period (15 days to <3 months), and late postoperative period (3 months to <12 months). All patients had a minimum follow-up of 12 months after the first surgery. Seizures were categorized in accordance with the International League Against Epilepsy operational classification.^21^ Engel classification was used to determine postoperative seizure freedom at 12 months.^22^

Findings were compared between 3 groups of patients: (i) preestablished ASMs group (patients that were prescribed ASMs at least 2 weeks before due to initial presentation with seizure or history of epilepsy); (ii) prophylactic ASMs group (patients prescribed ASMs prior to surgery as prophylaxis or commenced ASMs less than 2 weeks before surgery); and (iii) no ASM group (patients who were not on any ASMs until after their surgery).

Seizure control was defined as complete freedom from any epileptic seizures at the time of the last clinic review with or without ASM. Correlation between EOR and seizure rate in different time periods was compared between the 3 groups of patients. Differences between awake craniotomy technique and general anesthesia surgery were also compared in all 3 groups.

### Statistical Analyses

To determine the association between preoperative medications, EOR, awake/not and postoperative seizure, we ran 3 separate binary logistic regression models, each with its own set of carefully prespecified confounders. The confounding factors adjusted for included: tumor location, steroids presurgery, neuropathic medications, revision surgery, alcohol intake, Karnofsky score, medication history of immunosuppressants and autoimmune disorders. Due to their only being 13 patients in ASM group 2 (prophylactic), these were combined with those in ASM group 3 for the purpose of this analysis. We used descriptive statistics to show the frequency of postoperative seizure at each time point.

Un-adjusted curves for overall seizure-free survival were plotted using the Kaplan–Meier method, using log-rank tests to assess significance for group comparison.

Considering the retrospective and exploratory nature of this study, no power calculation had previously been performed, and no corrections for multiple statistical comparisons were made. Statistical significance was considered a 2-tailed *P* value of <.05. Statistical analyses were performed using Stata software version 14.0 (StataCorp).

### Ethical Approval

This retrospective case series was registered with the local tumor board and approved by the Oncology MDT.

### Study Limitations

Updated molecular factors were not used in this study for cases pre-2022 due to limited resources. Furthermore, only LGG cases from 2021/2022 onward were categorized using additional molecular characterization markers, such as CDKN2A/B.

## Results

A total of 206 patients underwent 257 operations (Table 1). Patients may have undergone 1, 2, or 3 operations due to recurrence/tumor progression.

**Table 1.** General Characteristics of the Study Population (*n* = 257)

Clinical Parameter	Category	*n*	%
Gender	Female	106	41.2
	Male	151	58.8
Age (mean (SD), years)	38.8 (12.2)	257	100.0
Main presenting symptom	Seizure	161	62.6
	Headache	48	18.7
	Focal neurology	9	3.5
	Behavioral changes	1	0.4
	Speech impairment	4	1.6
	Memory impairment	1	0.4
	Other^a^	33	12.8
Preop KPS	80–100	244	94.9
	50–70	13	5.1
Tumor location	Temporal	78	30.4
	Frontal	144	56.0
	Parietal	24	9.3
	Occipital	4	1.6
	Other^b^	7	2.7
Tumor side	Right	111	43.2
	Left	143	55.6
	Bilateral	3	1.2
Group	Established on ASM	189	73.5
	Prophylactic ASM	13	5.1
	Control (no ASM)	55	21.4
Surgical technique	Awake craniotomy	105	40.9
	General anesthesia	152	59.1
Type of ASMs (treatment group)	Valproate	63	33.3
	Levetiracetam	93	49.2
	Lamotrigine	22	11.6
	Phenytoin	4	2.1
	Other^c^	7	3.7
Seizure occurrence	Early (<15 days)	47	25.4
	Early–medium (15 days to 3 months)	57	30.8
	Medium–late (3–12 months)	81	43.8
Type of seizure	Generalized tonic–clonic	71	38.4
	Focal unaware	35	18.9
	Focal aware	79	42.7
Complications to AED	None	248	96.5
	Rash	3	1.2
	Lethargy	2	0.8
	Weight gain	1	0.4
	Tremor	1	0.4
	Memory disturbance	1	0.4
	Hair loss	1	0.4
Histology	Astrocytoma	135	52.5
	Oligodendroglioma	122	47.5

ASM, anti-seizure medication.

^a^Incidental.

^b^Brainstem, pontine, cerebellar.

^c^Clobazam, carbamazepine.

Of the 257 cases, 189 cases (73.5%) were preestablished on ASMs, 55 cases (21.4%) had no ASMs, and 13 cases (5.1%) were prescribed ASMs as prophylaxis. Originally, there were 19 cases in the prophylactic ASM group; however, 6 cases were moved to the preestablished ASMs, because treatment ASMs were started prior to prescribing a prophylactic ASM. Monotherapy ASMs used as prophylaxis were administered on the day of surgery and discontinued within 7 days if there was no evidence of seizure activity. Mean follow-up time was 5.77 years, ranging from 0.276 (3 months) to 16.6 years (Table 1).

Seizure was the main presenting symptom at diagnosis (62.6%) (Table 1). The frontal region was the most common LGG location (56.0%), followed by temporal (30.4%) and less frequently parietal or occipital (9.3% and 1.6%, respectively). Postoperative seizure frequency per location was highest in the temporal lobe (temporal, 50%; frontal, 45.1%; parietal, 33.3%; occipital, 25%; and other, 14.3%) (Table 1). Engel classification at 12 months postoperatively, 169 cases were grade 1 (65.8%), 41 cases grade 2 (16.0%), 23 cases grade 3 (8.9%), and 24 cases grade 4 (9.3%).^22^

ASMs were used as prophylaxis for 13 cases with no intraoperative or postoperative ASM-related complications. The most commonly used ASM in both prophylactic and treatment groups was levetiracetam (Table 1). Of the 257 cases, 215 cases were on 1 ASM agent (83.7%), 38 cases were on 2 ASM agents (14.8%), and 4 cases were on 3 or more ASM agents (1.5%). No major side effects were reported. A multivariate analysis was performed to determine the probability of a seizure with (treatment and prophylactic group) or without ASMs. After adjusting for prespecified confounders, the odds of seizure by 12 months postsurgery were increased for those on preoperative anticonvulsants, although the confidence interval is wide and the effect is not statistically significant (OR 2.13, 95% CI 1.00–4.54, *P* = .05) (Table 2).

**Table 2.** Logistic Regression Models to Determine If More or Less Likely to Have a Seizure Within 12 Months With Regards to the Following Independent Variables of Interest: Preop Anti-seizure Medications, Extent of Resection, and Awake Craniotomy Versus General Anesthetic

Outcome	Independent Variable of Interest	OR (95% CI)	*P* Value	Adjusted for
Seizure within 12 months	Preop anti-seizure medications	OR 2.13, 95% (CI 1.00–4.54)	.05	Tumor location, steroids presurgery, neuropathic medications, revision surgery, alcohol intake, Karnofsky score, medication history of immunosuppressants, and autoimmune disorders
Seizure within 12 months	Extent of resection			ASMs, tumor location, revision surgery, steroids, medication history of neuropathic, alcohol intake, and autoimmune diseases
	Total	Reference		
	Near-total	OR 5.5 (CI, 1.6–19.3)	.008	
	Subtotal-debulking	OR 7.1 (CI, 2.4–21.1)	<.001	
	Biopsy	OR 8.6 (CI, 2.0–36.1)	.003	
Seizure within 12 months	Awake/not	OR 1.4 (CI, 0.8–2.5)	.22	Tumor location and preop anticonvulsants

Overall, 114 cases experienced at least 1 or more seizure episodes (185 episodes in total) within 12 months of surgery. The mean time to seizure failure was 297.7 days postcraniotomy. Of the total seizure episodes, 25.4% occurred within 2 weeks of surgery, 30.8% between the timepoint of 2 weeks and 3 months and 43.8% between 3 months and 12 months. The most common seizure type was focal aware seizures at 44.1%, followed by generalized tonic–clonic at 36.7% and focal unaware at 19.2% (Table 1). Histologically, there were 135 cases (52.5%) of IDH-mutant grade 2 diffuse astrocytoma and 122 cases (47.5%) of IDH-mutant grade 2 oligodendroglioma, reclassified in accordance with the 2021 WHO classification.^19^ Of the 257 cases, preoperative seizure rate for astrocytoma was 73.3% (99/135), and oligodendroglioma was 73.8% (90/122). Postoperatively at 12 months, the seizure burden was higher with diffuse astrocytoma 48.9% (66/135) (and lower with oligodendroglioma, 45.1% (55/122)).

Awake craniotomy was performed in 105 cases (40.9%), with 84 (80.0%) out of 105 cases in the preestablished ASM group, 8/105 (7.6%) prophylactic ASM group and 13/105 (12.4%) no ASM group. Neurophysiology monitoring was used in 104 (99.0%) awake craniotomy cases and in 35 (23.0%) general anesthetic (GA) cases. A total of 19 intraoperative seizures occurred, 2 (10.5%) of which occurred under GA. Intraoperative seizures that occurred during awake craniotomy were subcategorized further: 6 (31.6%) occurred during awake throughout surgery and 11 (57.9%) during GA/awake surgery. In order to determine the association between awake or GA and postoperative seizure, we performed a multivariate analysis. Results showed there is no evidence of an association between being awake or not and seizures at 12 months postoperatively (OR 1.4, 95% CI 0.8–2.5, *P* = .22), after adjusting for prespecified confounders: tumor location and preop anticonvulsants (Table 2).

The EOR was inversely related to seizure activity at all timepoints postoperatively. Overall, 32 cases (12.5%) were GTR, and 33 cases (12.8%) were NTR (Table 3). In total, 18 cases were converted from an awake craniotomy to GA or discontinued due to impending neurological deficits. Of these 18 cases discontinued or converted to GA; GTR was achieved in 3 cases (16.7%), a further 3 cases (16.7%) achieved NTR, 11 cases (61.1%) achieved STR and only 1 case (5.6%) achieved a biopsy only. Postoperatively, 9 (3.5%) patients suffered with a permanent deficit (hemiparesis, speech disturbance, or visual disturbance) (Table 4). In the early postoperative period (<15 days), seizure failure was calculated from the total cases per EOR type. Results showed that there was a reduction in postoperative seizures with GTR compared to all other EOR types (GTR, 3.1%; NTR, 18.1%; STR, 18.7%; and biopsy, 26.9%) (Table 3). A further multivariable analysis was performed to investigate the association between EOR and seizure outcome. After adjusting for prespecified confounders (ASMs, tumor location, revision surgery, steroids, medication history of neuropathic, alcohol intake, and autoimmune diseases), near-total, subtotal, and biopsy surgical resection types were associated with a statistically significant increase in odds of seizure by 12 months postoperatively (respective ORs [95% CIs]: 5.5 [1.6–19.3], *P* = .008, 7.1 [2.4–21.1], *P* < .001, 8.6 [2.0–36.1], *P* = .003), compared with GTR (Table 2).

**Table 3.** Extent of Resection Versus Seizure Failure at 4 Different Timepoints (15 Days; 15 Days to 3 Months, 3–12 Months, and >12 Months)

Extent of Resection (EOR) Type	Seizure <15 Days	Seizure 15 Days to 3 Months	Seizure 3–12 Months	Seizure >12 Months
Gross-total (100%)	1 (3.1%)	2 (6.3%)	3 (9.4%)	7 (21.9%)
Near-total (>90%)	6 (18.1%)	3 (9.1%)	10 (30.3%)	7 (21.2%)
Subtotal (50%–90%)	31 (18.7%)	39 (23.5%)	59 (35.5%)	74 (44.6%)
Biopsy	7 (26.9%)	8 (30.8%)	8 (30.8%)	9 (34.6%)

Patients who suffered with seizures may have suffered with seizures at more than 1 timepoint. Number of EOR cases: gross-total resection 32, near-total 33, subtotal 166, and biopsy 26.

**Table 4.** Surgical Complications Recorded Intraoperatively and Postoperatively

Complication	Postop Surgical Complications	Transient Neurosurgical Outcomes
Cerebral swelling/hydrocephalus	4	—
Hemorrhage	4	—
Expressive dysphasia	26	24 (92.3%)
Receptive dysphasia	1	1 (100%)
Hemiparesis	16	11 (68.8%)
Wound infection	5	—
Intracranial infection	12	—
Facial weakness	7	7 (100%)
Fine motor coordination issues	1	1
Pseudomeningocoele	12	—
Cognitive deficit	7	7 (100%)
Visual disturbances	2	0 (0%)
Sensory deficit	1	1 (100%)

Intraop, intraoperative; Postop, postoperative. Transient neurological outcomes recovered within 6 weeks from surgery.

## Discussion

In this study, we report our center’s experience in treating and managing LGG-related epilepsy. All cases were performed by subspecialist Consultant Neuro-Oncology surgeons. During the period of this study, we had an increasing number of awake craniotomies due to expansion of our service. From 2007 to 2015, 37 cases underwent an awake craniotomy with intraoperative mapping (IOM), while 67 awake cases with IOM were performed from 2016 to 2022. In GA cases, only 4 cases pre-2016 used IOM, in contrast to 31 cases from 2016 onward. Our ratio of awake versus GA craniotomy changed from 38:86 cases pre-2016 to 67:69 cases post-2016. Our results also showed a trend toward higher GTR outcomes in awake craniotomy cases, 18 versus 14 GA cases. However, the awake craniotomy cases were associated with higher rates of postoperative seizures when compared with GA, but with no significant statistical finding at 12 months. Previous studies have reported similar findings.^23,24^

In our study population, we were not able to statistically assess prophylactic treatment with ASMs before surgery due to the small sample size; however, observational data did not demonstrate any benefits in reducing early postoperative seizure rates. This could be explained by either insufficient loading time to allow ASMs to work or selection bias, considering patients thought to be at a higher risk for seizure would more likely fall in the prophylactic ASMs group. Overall, our postoperative seizure rate was 45/257 (17.5%) at <15 days, 52/257 (20.2%) at 15 days to 3 months and 76/257 (31.1%) at 3–12 months. After 12 months, 97/257 (37.8%) cases continued to have seizures with the remaining patients maintaining seizure freedom or under control with medications. Other studies have found varying results with higher and lower seizure outcomes.^5,24–26^ Further histological subtype analysis showed a lower, but not significant postoperative seizure rate with grade 2 oligodendroglioma, 45.1% (preoperative: 73.8%), versus grade 2 astrocytoma patients 48.9% (preoperative: 73.3%). Carstam et al. noted a similar frequency of postoperative seizures in both groups but a slightly higher rate with oligodendroglioma.^18^ The slight variation could be due to a number of factors that include EOR, tumor location, ASM use, and postoperative morbidity.

ASM use at the time of cranial surgery was initially shown to be of benefit by North et al.^10^; on the contrary, a meta-analysis showed that ASMs did not significantly decrease seizure rate.^27^ Recent studies have focused on the type of ASM used with increasing benefits of seizure management using second-generation ASMs and reduced adverse effects in comparison to older anticonvulsants.^14,28^ Nonetheless, the overall benefits versus the risks of using ASMs prophylactically remain an ongoing debate. The American Academy of Neurology in 2000 recommended discontinuing prophylactic ASMs within 1 week of starting medication in seizure-naïve patients postsurgery, but studies report varying adherence.^29^ More recently, SNO and EANO guidelines have proposed that no ASMs should be given prophylactically.^12^ Our practice shows that only 13 patients were given prophylactic ASMs, which were discontinued within 1 week, while the remaining cases were either on treatment or no ASM. Conclusively, there is no evidence from our multivariate analysis to support the use of ASMs prophylactically. However, one may suggest that such a blanket statement negates the management of the individual patient at epileptogenic risk. Seizures can lead to a significant impact on the patient’s neurocognition and quality of life, such as daily activities, driving ability and overall safety.^30,31^ Therefore, identifying prognostic markers of risk or predictive value for patients more likely to develop seizures would benefit patient management and structure the use of ASMs.

One definitive prognostic marker for postoperative seizure control is EOR. Studies have shown the universal benefits of seizure freedom postresection.^25^ However, the total volume resection benefit has shifted from >80% resection,^25^ to GTR, which demonstrated benefits to seizure management and overall survival.^20,32^ Our center aims for GTR in all cases within the confounds of tumor location, neurology preservation, and patient tolerance. Our results, in line with the literature, showed a reduction in seizures when comparing GTR versus STR.^33^ Borger et al. recommended performing a GTR and anterior temporal lobectomy for temporal lobe high-grade gliomas, with results showing 100% seizure freedom in 13 patients.^17^ Furthermore, some units suggest that a supra-total resection in LGGs improves survival and reduces seizure frequency.^34,35^ Nonetheless, the EOR should be judged on an individual basis and the nature of the histology. For instance, it is suggested that EOR is of higher importance in diffuse astrocytoma to prevent early transformation and progression.^36^ On the contrary, the potential neurological deficits and long-term disability are at a higher risk for patients’ postradical surgery. Thus, a *carte blanche* approach of supra-total resection for all is not a currently supported approach with no randomized controlled trials or prospective studies investigating the benefits.^37^

Currently, there is no level 1 evidence supporting the choice of ASMs for the treatment of seizures in patients with brain tumors. Newer ASMs have recently become the preferred first choice in our center due to their limited enzyme-inducing profile, prevalent renal excretion, lower plasma protein binding, and, consequently, fewer interactions with chemotherapeutic agents.^38,39^ Combining ASMs with the latest surgical techniques, trends toward an overall improved reduction in seizure frequency and overall survival. Therefore, the decision to prescribe prophylactic ASMs should be judged on an individual basis, with potential nonstatistically significant benefits to certain patients.

Due to its retrospective nature, our study has some limitations. The authors acknowledge that the majority of the patients in the prophylaxis group underwent an awake craniotomy, which in itself could have a greater predisposition to intraoperative seizures due to the cortical stimulation.^13,40^ Despite this, our findings showed no significant difference in awake versus not with respect to seizure outcome in the postoperative period. This finding could be tested further in the context of a prospective randomized controlled trial. Furthermore, we included patients treated from 2006 with older generation ASMs, while surgery from 2016 onward used newer generation ASMs, thus limiting comparisons regarding ASM efficacy. Finally, data regarding seizure management with impact on quality of life were not available.

## Conclusions

In line with the current literature, in this large case series of LGG patients, we have shown that the location and extent of surgical resection were predictive factors of postoperative seizures. Interestingly, gliomas of the temporal region had a higher incidence of seizure, independent of ASM use. In our study, patients on preexisting ASMs have a higher chance of postoperative seizures, but we cannot definitively comment on the prescription of prophylactic ASMs. Currently, the routine use of prophylactic ASM is not widely recommended; however, subgroups of patients with prognostic indicators suggesting a higher risk of seizures might benefit from the prophylactic use of ASM. In conclusion, a larger multicenter study with longer follow-up might be required to confirm our results.
